# Comparison of Discectomy versus Sequestrectomy in Lumbar Disc Herniation: A Meta-Analysis of Comparative Studies

**DOI:** 10.1371/journal.pone.0121816

**Published:** 2015-03-27

**Authors:** Jisheng Ran, Yejun Hu, Zefeng Zheng, Ting Zhu, Huawei Zheng, Yibiao Jing, Kan Xu

**Affiliations:** Department of Orthopedic Surgery, 2nd Affiliated Hospital, School of Medicine, Zhejiang University, Hangzhou, China; University of Michigan, UNITED STATES

## Abstract

**Background:**

Lumbar disc removal is currently the standard treatment for lumbar disc herniation. No consensus has been achieved whether aggressive disc resection with curettage (discectomy) versus conservative removal of the offending disc fragment alone (sequestrectomy) provides better outcomes. This study aims to compare the reherniation rate and clinical outcomes between discectomy and sequestrectomy by literature review and a meta-analysis.

**Methods:**

A systematic search of PubMed, Medline, Embase and the Cochrane Library was performed up to June 1, 2014. Outcomes of interest assessing the two techniques included demographic and clinical baseline characteristics, perioperative variables, complications, recurrent herniation rate and post-operative functional outcomes.

**Results:**

Twelve eligible trials evaluating discectomy vs sequestrectomy were identified including one randomized controlled study, five prospective and six retrospective comparative studies. By contrast to discectomy, sequestrectomy was associated with significantly less operative time (p<0.001), lower visual analogue scale (VAS) for low back pain (p<0.05), less post-operative analgesic usage (p<0.05) and better patients’ satisfaction (p<0.05). Recurrent herniation rate, reoperation rate, intraoperative blood loss, hospitalization duration and VAS for sciatica were without significant difference.

**Conclusions:**

According to our pooled data, sequestrectomy entails equivalent reherniation rate and complications compared with discectomy but maintains a lower incidence of recurrent low back pain and higher satisfactory rate. High-quality prospective randomized controlled trials are needed to firmly assess these two procedures.

## Introduction

Lumbar disc herniation (LDH), mainly presenting low back pain and radiculopathy, is a common condition to those who needs spine surgeons. The incidence of LDH is reported as 1% to 2% in general population [1,2] and 4.86 per 1000 person-years in young population [3]. Most LDH patients will improve independent of treatment or through conservative treatment such as spinal manipulation, epidural steroid injections, structured exercise and etc [4]. Nevertheless, for patients who are refractory to conservative therapies, surgical intervention is recommended [4,5].

Since Mixter and Barr [6] finished the first successful lumbar herniated disc resection involving extensive lamina removal in 1934, less invasive approaches had being developed in lumbar disc surgery. Among which, two methods have been debated over the past decades. One is discectomy, introduced by O’Connell [7], including aggressive curettage of the normal disc as well as removal of the offending herniated disc fragment. The concept of this procedure is that the remained disc has a high incidence of reherniation and subsequently causes recurrence of symptoms. However, the curettage of disc space leads to collapse of disc height, which gives rise to intervertebral instability and accelerate spondylosis [8], thus contributing to the “failed back syndrome” [9]. The other is sequestrectomy, described by Williams [10] and Spengler [11], consisting of removal of the disc fragment alone without or with little invasion of the disc space. Benefiting from the retention of normal disc and endplates, this conservative procedure is considered to retain disc height and minimally disturb the intervertebral instability.

Both of the two procedures are widely used in clinical practice, but it still reaches no consensus which provides the best long-term outcome. The aim of the present study is to compare the complications and clinical outcomes between the two procedures by reviewing literature and a meta-analysis.

## Methods

We have conducted this review in accordance with the PRISMA guidelines [12]. The checklist is provided in S1 PRISMA Checklist.

### Search strategy

In the absence of large well-designed prospective randomized controlled trials (RCTs), both RCTs and non-RCT comparative studies which compared discectomy with sequestectomy were included. We searched databases including PubMed, MEDLINE, EMBASE and Cochrane Central Register of Controlled Trials up to June 1, 2014. The search strategy consist of a combination of keywords concerning the sequestrectomy related terms (sequestrectomy OR herniotomy OR fragmentectomy OR "fragment excision" OR "limited discectomy" OR "limited microdiscectomy"), the discectomy related terms (discectomy OR microdiscectomy) and the anatomical terms (lumbar vertebrae). The research was limited to English publications. The eligibility criteria were applied: 1) the study design was comparative (discectomy versus sequestrectomy). 2) The study population was composed of patients older than 15years who were diagnosed as lumbar disc herniation and refractory to conservative treatments. 3) At least one of the following data was presented: operative time, intra-operative blood loss, duration of hospitalization, perioperative complications, Visual Analogue Scale (VAS), analgesic administration, satisfactory rate, reherniation rate and reoperation rate. 4) The follow-up time was no less than 1 year. The excluded criteria was as followed: 1) the study population consisted of patients with a history of spine surgery at the same level or with notable nonintervertebral disc abnormalities, such as spondylolysis, spondylolisthesis, inflammatory arthritis or metabolic bone disease. 2) The outcomes were compared within patients from different medical centers. 3) Repeated studies. Two investigators (JS.R and YJ.H) checked all titles, abstracts and full publications searched from database independently. If inconsistencies occurred between the two investigators, a discussion was carried out until a consensus was reached.

### Data extraction

Two investigators (JS.R and YJ.H) reviewed full publications and extracted data as followed independently: 1) basic information of included studies, consisting of study type, country, study year, enrolled number and follow-up time. 2) Baseline characteristics of study population, including age, sex, duration of symptoms before surgery, body mass index (BMI), diagnosis, level proportion, comorbidity and smoke. 3) Surgical and perioperative information, including operative time, intra-operative blood loss, duration of hospitalization and complications. 4) Clinical outcomes, composed of reherniation rate, reoperation rate, VAS for low back pain and sciatica at the last evaluation time, analgesic administration in both <1year and >1 year post operation separately and patients’ satisfaction rate. Long-term outcomes (Reherniation rate, VAS for low back pain and sciatica) of Thome,C’s [13] study were extracted in a subsequent publication [14]. A standardized form was used and any discrepancies were resolved by discussion.

### Study quality

Two investigators (JS.R and YJ.H) appraised the quality of each included study independently by two different assessing tools for RCTs or non-RCTs. For RCTs, Detsky quality index [15] was utilized with a maximum score of 20 for positive trials and 21 for negative trials respectively. For non-RCTs, the MINORS score [16] was applied with a total score of 24. A study whose score was more than 75% of the maximum score was considered high quality.

### Statistical analysis

Meta-analyses were performed either using risk ratio (RR) or weighted mean difference (WMD) with 95% confidence intervals (CIs) as summary of statistic for binary or continuous variables, respectively. The analyses were performed under guiding of the recommendations of the Cochrane Collaboration and the Quality of Reporting of Meta-analyses guidelines [17]. Random effects models were applied if the heterogeneity among studies was significant by using the χ^2^ test and Ι^2^ statistics, otherwise the fixed effect models were utilized. Statistical significance was set as p<0.05. Data were analyzed using Review Manager version 5.3 (The Cochrane Collaboration, Oxford, UK).

## Results

**Fig 1:**
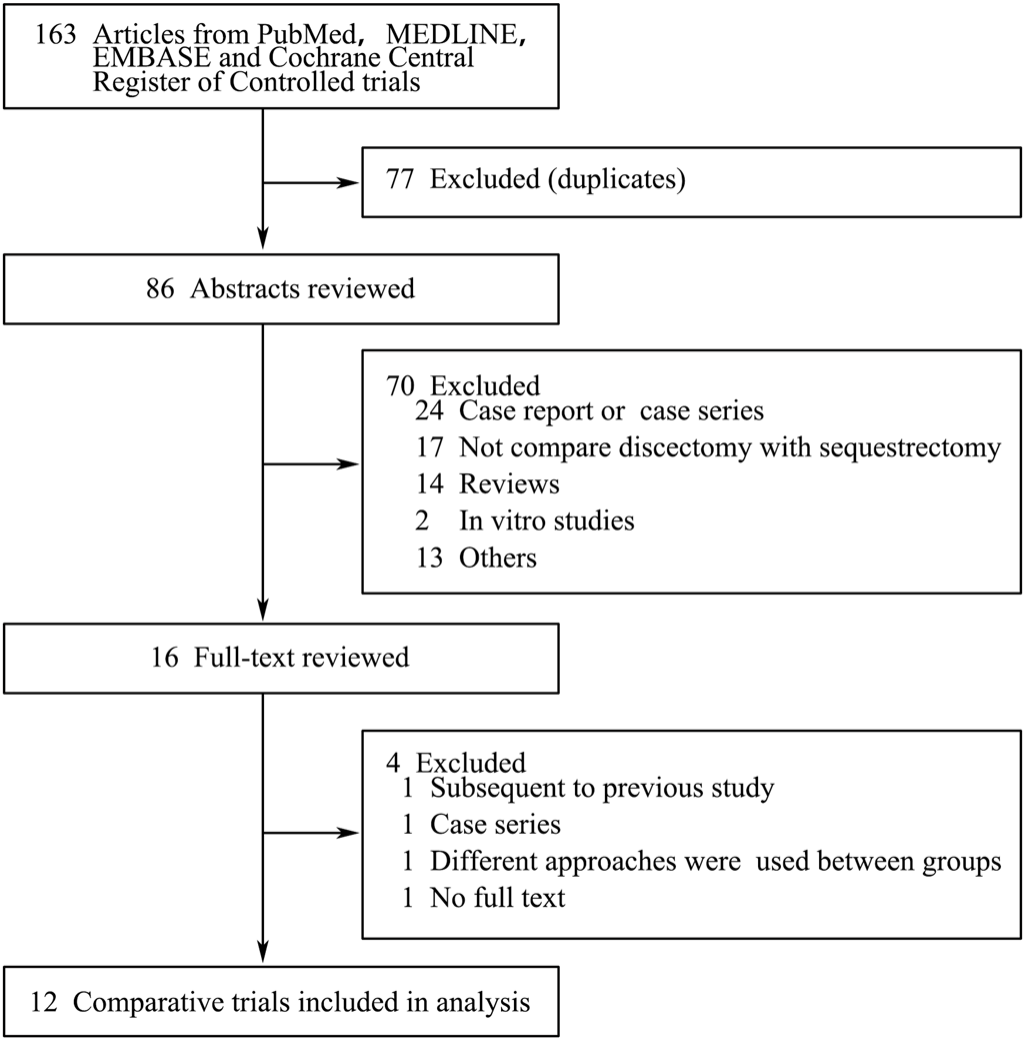
Flow diagram of the literature search.

According to the searching strategy illustrated in Fig. 1, 12 eligible comparative studies were identified, including one randomized controlled trial [13], 5 prospective comparative studies [18–22] and 6 retrospective comparative studies [23–28]. Two [18,22] of the 12 studies were historical comparative and sequestrectomy was prior to discectomy, other studies all consisted of contemporary groups. Four highly relevant publications were excluded: one was a long-time follow-up subsequent to a previous study [14]; one was a case series study [11]; one applied different surgical approaches between sequestrectomy and discectomy [29]; one was lack of full text [30]. A total of 1648 participants were reviewed, of which 900 were in discectomy group and the rest 748 were in sequestrectomy group.

### Study characteristics

The brief description of the included studies was listed in Table 1. According to the study quality assessment, there were four high quality studies and eight low quality studies. The mean follow-up time ranged from 12 months to 62.4 months. Baseline comparisons were also performed in all included studies. All participants were diagnosed with lumbar disc herniation by symptoms and image evidence. However, pathologic level distribution and gender differed significantly in two studies and as to symptoms duration, comorbidity and smoke, significant difference was reported in one study. Other baseline characteristics comparison was either statistically insignificant or not available (Table 2).

**Table 1:** Brief description of the included 12 studies.

Study	Years	Country	Study design	Quality scale^a^	No. of patients (D:S)	Mean follow up (mo)	Mean age (y) (D:S)	Gender (% Male) (D:S)
Rogers, L.A et al	1988	USA	PCS	14/24	35:33	11–30	43.4:45.5	45.7:66.7
Faulhauer, K et al	1995	Germany	RCS	13/24	100:100	42.7	44.8:51.9	57.0:66.0
Thome, C et al	2005	Germany	RCT	17/21	42:42	12–18	40.0:42.0	54.7:57.1
Carragee, E. J et al	2006	USA	PCS	15/24	30:46	24.0	38.4:37.5	53.3:54.3
Kast, E et al	2008	Germany	PCS	18/24	88:80	24.0	41.9:45.4	58.0:58.8
Schick, U et al	2009	Germany	PCS	16/24	100:100	34.0	52.8:49.5	64.0:50.0
Fakouri, B et al	2011	UK	RCS	18/24	72:24	32.7	38.4:37.2	63.9:62.5
Baek, G. S et al	2012	Korea	RCS	14/24	101:74	23.2	48.3:42.9	59.4:54.1
Park, J. S et al	2013	Korea	RCS	16/24	57:57	14.4	47.6:50.0	57.9:54.4
Shamji, M. F et al	2013	Canada	RCS	17/24	98:74	60.0	44.1:44.4	63.0:64.0
Kotil, K et al	2014	Turkey	RCS	16/24	85:40	62.4	41.4:39.9	43.5:47.5
Boyaci, S et al	2014	Turkey	PCS	19/24	92:78	34.8	46.2:45.3	47.8:52.6

RCS retrospective comparative study, PCS prospective comparative study, RCT randomized controlled study

^a^ RCT was assessed by Detsky score and non-RCT was assessed by MINORS score.

**Table 2:** Comparison of baseline characteristics in each included study.

Study	Years	Age	Gender	Symptoms duration	BMI	Diagnosis	Level	Comorbidity	Smoke
Rogers, L.A et al	1988	#	*	NA	NA	NA	NA	NA	NA
Faulhauer, K et al	1995	#	#	NA	NA	#	*	NA	NA
Thome, C et al	2005	#	#	#	#	#	#	NA	NA
Carragee, E. J et al	2006	#	#	#	NA	#	NA	NA	NA
Kast, E et al	2008	#	#	NA	NA	#	NA	NA	NA
Schick, U et al	2009	#	*	*	NA	#	*	*	NA
Fakouri, B et al	2011	#	#	NA	NA	#	#	NA	NA
Baek, G. S et al	2012	#	#	NA	NA	#	#	NA	NA
Park, J. S et al	2013	#	#	NA	NA	#	#	NA	NA
Shamji, M. F et al	2013	#	#	NA	#	#	#	NA	*
Kotil, K et al	2014	#	#	NA	NA	#	#	NA	NA
Boyaci, S et al	2014	#	#	NA	#	#	#	NA	#

* statistically significant.

^#^ statistically insignificant.

NA not available.

### Operative time and intraoperative blood loss

**Fig 2:**
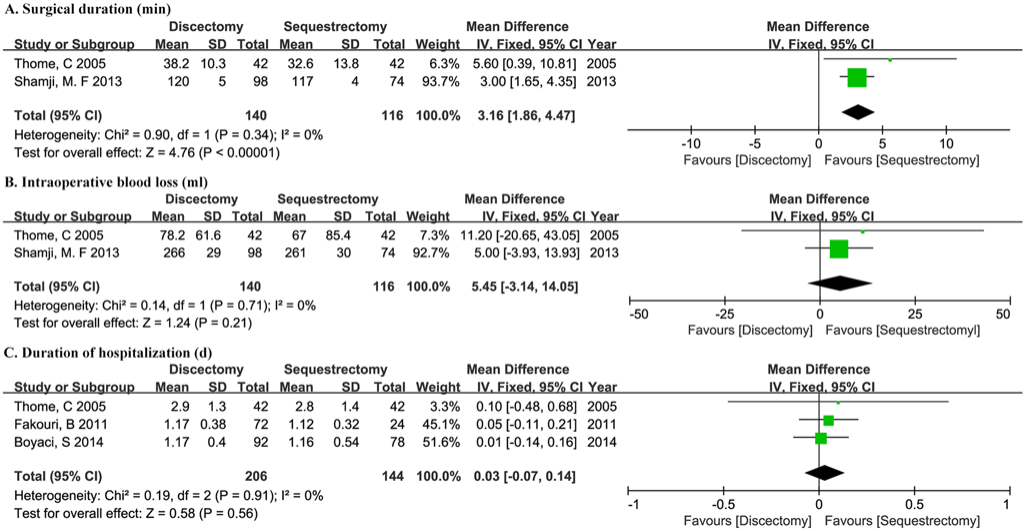
Forest plot illustrating operative time (A), intraoperative blood loss (B), hospitalization duration (C) of meta-analysis comparing discectomy with sequestrectomy.

Adequate data with the mean and standard deviation (SD) regarding operative time and intraoperative blood loss were reported in two studies [13,27]. Single mean values without SD of operative time were provided by four studies [19,24,25,28], all of which presented shorter operative time in sequestrectomy group. The two included studies enrolled 256 patients, with 140 patients assigned to discectomy group and the other 116 patients assigned to sequestrectomy group. The weighted mean difference of operative time is 3.16 (95%CI 1.86–4.47,P<0.001) in favor of sequestrectomy group. There’s no significant difference of intraoperative blood loss between the two groups (WMD 5.45, 95%CI -3.14–14.05, P = 0.21). No significant heterogeneity existed between studies (P = 0.34, *I* ^2^ = 0%; P = 0.71, *I* ^2^ = 0%; respectively) (Fig. 2A, B).

### Duration of hospitalization

Adequate data with mean and SD regarding hospitalization duration were provided in three studies [13,21,24], which enrolled 350 patients with 206 in discectomy group and 144 in sequestrectomy group. Single mean values without SD were provided by three studies, two of which reported shorter hospitalization in sequestectomy [20,28] and one of which reported no significant difference [27]. Heterogeneity test showed statistically insignificant (P = 0.91, *I* ^2^ = 0%). The weighted mean difference is equivalent between the two groups (WMD 0.03, 95%CI -0.07–0.14, P = 0.56) (Fig. 2C).

### Complications

**Fig 3:**
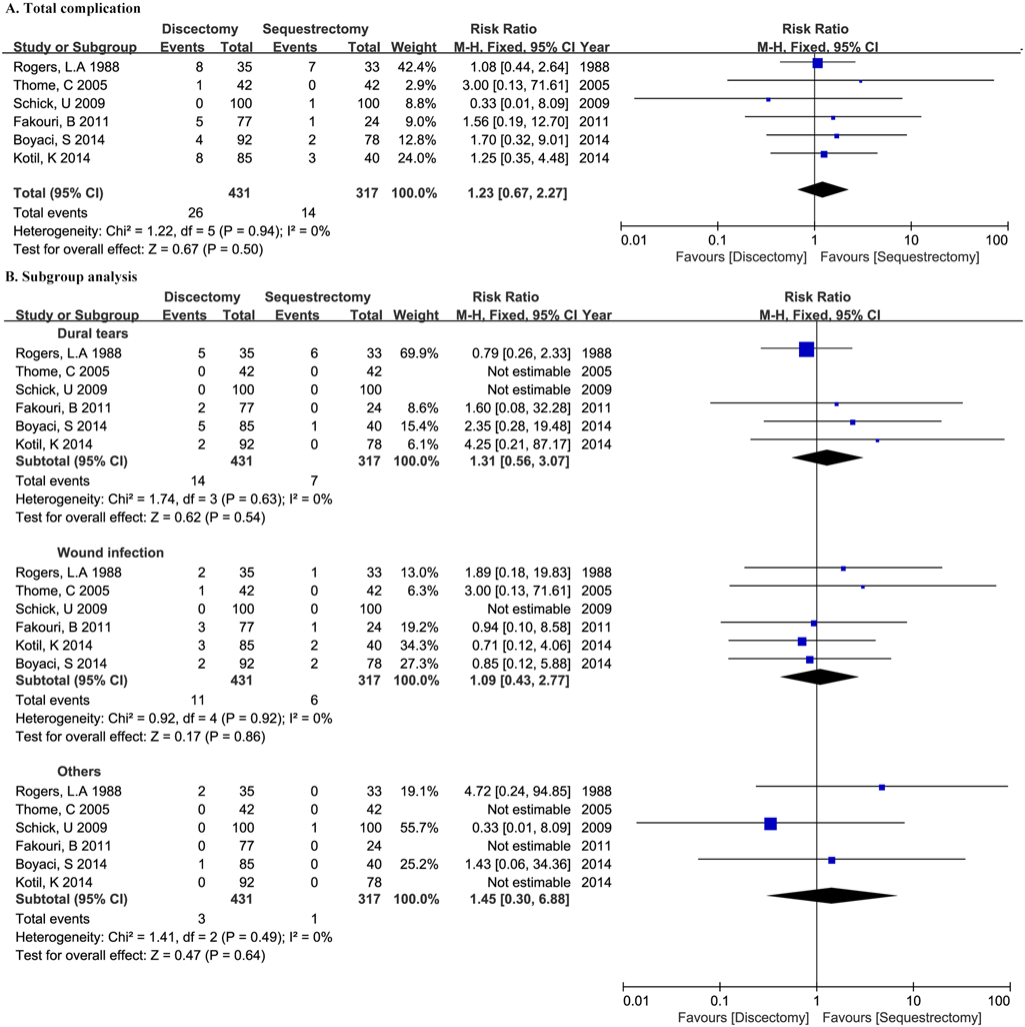
Forest plot illustrating total complication rate (A) and subgroup analysis (B) of meta-analysis comparing discectomy with sequestrectomy.

Complications were reported in six studies [13,20–22,24,28]. 748 patients were enrolled, with 431 patients and 317 patients assigned to discectomy group and sequestrectomy group, respectively. Meta-analysis demonstrated no significant difference between two groups (RR 1.23, 95%CI 0.67–2.27, P = 0.50). Heterogeneity was detected insignificant among groups (P = 0.94, *I* ^2^ = 0%) (Fig. 3A).

Reported complications mainly included dural tears and wound infection. Other complications include one epidural hematoma, one pseudomeningocele and one nerve root injury in discectomy group and one epidural hemorrhage in sequestrectomy group. None of these complication rates were confirmed significant different (RR 1.31, 95%CI 0.56–3.07, P = 0.54; RR 1.09, 95%CI 0.43–2.77, P = 0.86; RR 1.45, 95%CI 0.30–6.88, P = 0.64; respectively). There’s no significant heterogeneity among groups (P = 0.63, *I* ^2^ = 0%; P = 0.92, *I* ^2^ = 0%; P = 0.49, *I* ^2^ = 0%; respectively) (Fig. 3B).

### Reherniation

**Fig 4:**
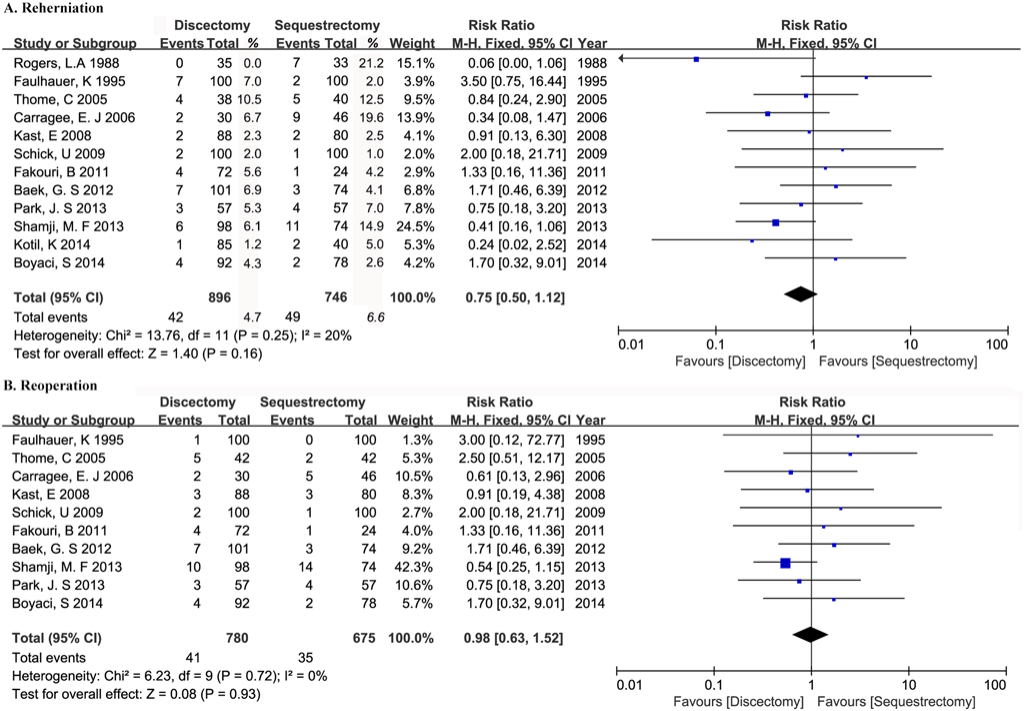
Forest plot illustrating reherinaiton rate (A) and reoperation rate (B) of meta-analysis comparing discectomy with sequestrectomy.

Twelve studies reported reherniation rate for at least one year follow up [13,18–28]. 1642 patients were enrolled, with 896 patients assigned to discectomy group and 746 patients assigned to sequestrectomy group. The reherniation rate in discectomy group ranged from 0% to 10.5% with an average of 4.7%, while that in sequestrectomy group ranged from 1.0% to 21.2% with an average of 6.6%. The two historical studies [18,22] contributed to the highest two reherniation rate in sequestrectomy group (21.2% and 19.6%, respectively). Though there’s a trend towards higher recurrent herniation rate in sequestrectomy group, our meta-analysis indicated no significant difference (RR 0.75, 95%CI 0.50–1.12, P = 0.16). No significant heterogeneity was detected among groups (P = 0.25, *I* ^2^ = 20%) (Fig. 4A).

### Reoperation

Reoperation rate was reported in ten studies [13,18–21,23–27]. They enrolled 1455 patients, with 780 patients in discectomy group and 675 patients in sequestrectomy group. Meta-analysis demonstrated no significant difference between the two groups (RR 0.98, 95%CI 0.63–1.52, P = 0.93). No significant heterogeneity was detected (P = 0.72, *I* ^2^ = 0%) (Fig. 4B).

### Visual analogue scale

**Fig 5:**
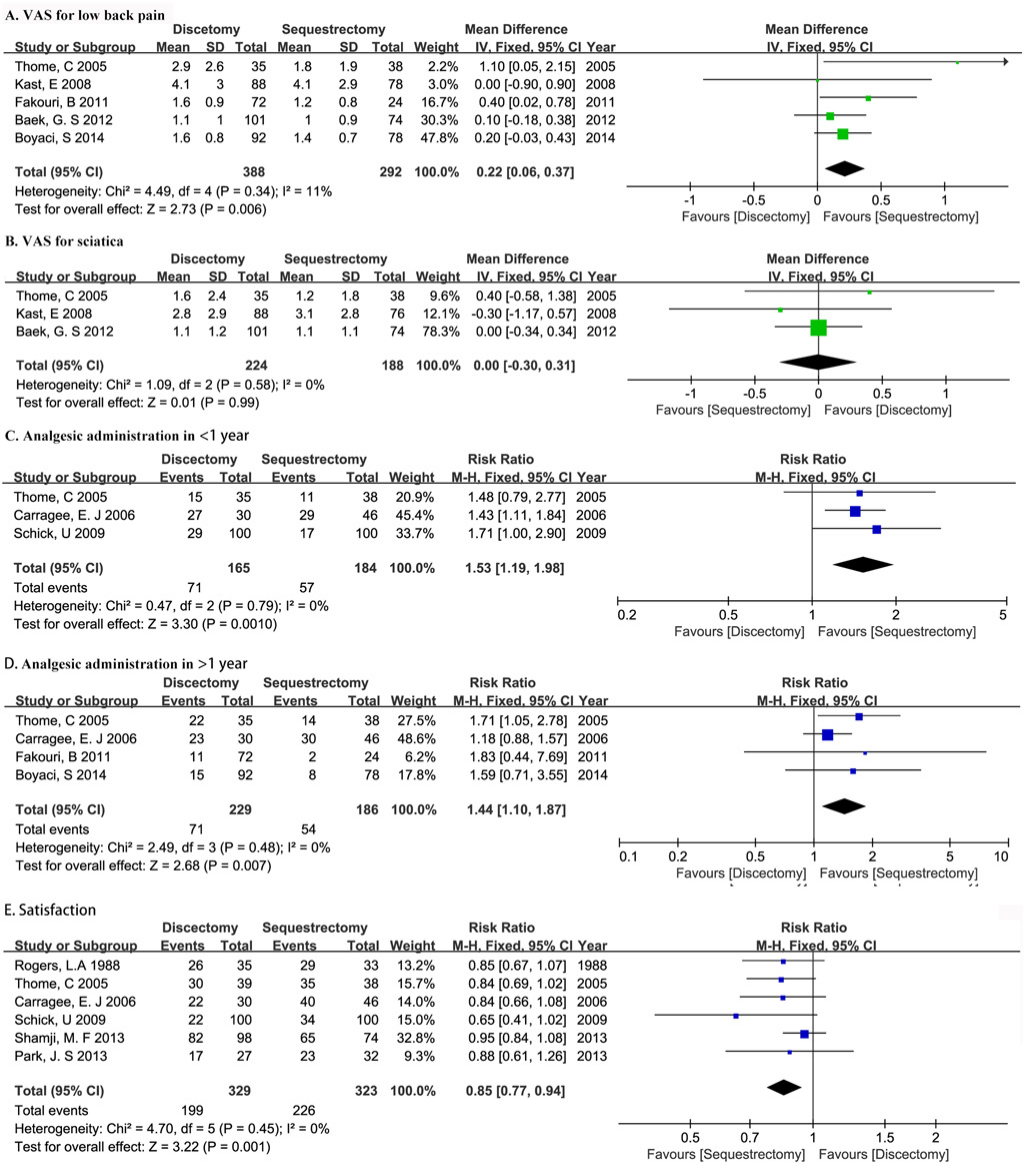
Forest illustrating plot post-operative VAS for low back pain (A), post-operative VAS for sciatica (B), analgesic administration in less than 1 year post operation(C), analgesic administration in more than 1 year post operation(D), and satisfaction rate (E) of meta-analysis comparing discectomy with sequestrectomy.

Adequate data of post-operative VAS for low back pain with mean and SD were provided in five studies [13,19,21,24,25], which enrolled 680 patients with 388 in discectomy group and 292 in sequestrectomy group. Four studies [18,20,26,28] provided single mean values without SD and reported no significant difference. Our meta-analysis revealed that post-operative VAS for low back pain favored sequestrectomy with a weighted mean difference of 0.22 (95% CI 0.06–0.37, P<0.05). No significant heterogeneity was detected (P = 0.34, *I* ^2^ = 11%) (Fig. 5A).

Three studies provided adequate data of post-operative VAS for sciatica with mean and SD [13,19,25], which enrolled 412 patients with 224 in discectomy group and 188 in sequestrectomy group. Three studies provided single mean values without SD [18,20,28], all of which showed no significant difference. Postoperative VAS for sciatica was equal between two procedures (WMD 0.00, 95%CI -0.30–0.31, P = 0.99). No significant heterogeneity was detected (P = 0.58, *I* ^2^ = 0%) (Fig. 5B).

### Analgesic administration

Analgesic administration rate in less than one year post operation was provided in three studies [13,18,20] and that in more than one year was reported in four studies [13,18,21,24], which enrolled 349 patients (165 in discectomy group and 184 in sequestrectomy group) and 415 patients (229 in discectomy group and 186 in sequestrectomy group), respectively. Heterogeneity was proved insignificant (P = 0.79, *I* ^2^ = 0%; P = 0.48, *I* ^2^ = 0%; respectively). Analgesic administration in both less than and more than one year favored sequestrectomy group (RR 1.53, 95%CI 1.19–1.98,P<0.05; RR 1.44, 95%CI 1.10–1.87,P<0.05; respectively) (Fig. 5C, D).

### Satisfaction

Six studies apprised post operation satisfaction [13,18,20,22,26,27], enrolling 652 patients with 329 in discectomy group and 323 in sequestrectomy group. No significant heterogeneity was found (P = 0.45, *I* ^2^ = 0%). The relative ratio was 0.85 (95%CI 0.77–0.94,P<0.05) in favor of sequestrectomy (Fig. 5E).

### Publication bias and sensitivity analysis

**Fig 6:**
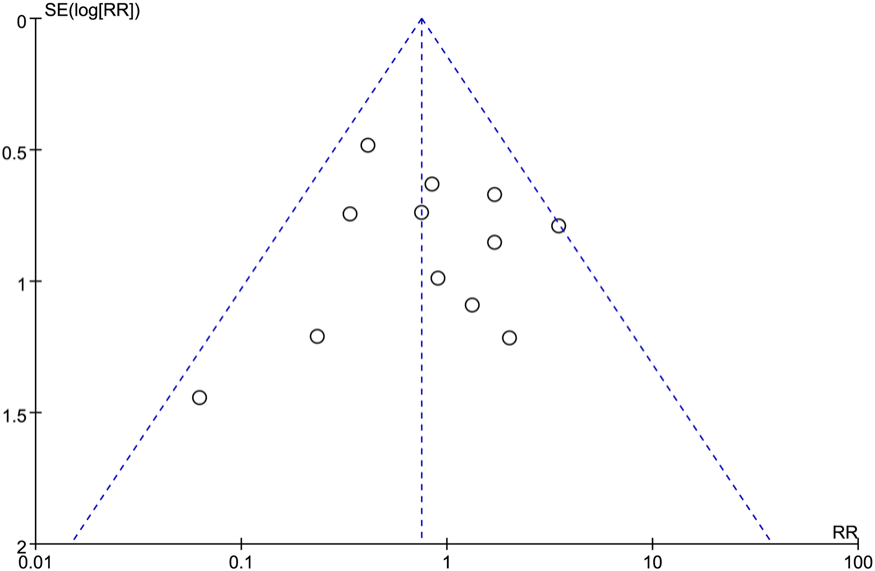
Funnel plot of reherniation rate.

The studies that reported reherniation rate had a fairly symmetrical distribution in the funnel plot. All studies were scattered within the 95%CI and spread evenly on both sides of the average, indicating little publication bias (Fig. 6). Sensitivity analysis was accomplished by reanalyzing our data after sequential eliminating individual study. Pooled results didn’t change significantly by eliminating any single study.

## Discussion

In this pooled study of around 1600 participants from 12 comparative studies, sequestrectomy was associated with less operative time, lower post-operative VAS for low back pain, less postoperative analgesic administration and higher satisfactory rate but equivalent reherniation rate, complications, reoperation rate, intraoperative blood loss, hospitalization duration and post-operative VAS for sciatica, by contrast to discectomy.

In need of the intervertebral space entrance and curettage, discectomy required significantly longer operative time than sequestrectomy. This pooled data was made out by including two studies and consistent results were also confirmed by four studies that provided single mean values without SD. Though total complications were statistically insignificant, a tendency toward higher incidence of complications existed in discectomy (RR = 1.23), which was especially notable in dural tears (RR = 1.31). In addition, intraoperative blood loss and hospitalization duration were also compared, resulting in no significant difference.

Disaccording to previous review literature [31,32] in 2009 which showed higher incidence of reherniation after sequestrectomy, an equivalent reherniation rate between discectomy and sequestrectomy was revealed in our meta-analysis and consistent with the result of another review conducted by Fakouri B et al [33] in 2014. The difference may be partially ascribed to the inclusion of low-evidenced non-comparative studies in both two reviews in 2009, which may result in bias for lack of appropriate control to eliminate the influence of surgeon’s experience and patients’ selection. Moreover, learning curve of surgeons was also demonstrated to affect prognosis and complications in endoscopic lumbar surgery [34,35]. The highest two reherniation rates after sequestrectomy ever were reported by Rogers, L.A et al [22] and Carragee, E.J et al [18] (21% and 19.6%, respectively), far more than other studies whose results were mainly below 10% (Fig. 4). Endoscopic technique was applied and sequestrectomy group was prior to discectomy group in time in both studies. Thus, the proficiency of endoscopic technique may influence the final outcomes in these two historical comparative studies. Similar conditions may also exist in other non-comparative studies. In addition, annulus fibrosis competence was crucial in lumbar disc surgery, fewer reherniation rate was reported when the annulus defect was less than 6 mm [18], and a decreased recurrence rate was observed when annular repair was utilized after microdiscectomy [36]. Only four of our included studies [18,22,23,27] reported an annular incision in sequestrectomy group and three of them [18,22,27] were relevant to relatively high reherniation rate (21%, 19.6%, 14.9%, respectively). On this occasion, the better annular protection may contribute to the relatively low reherniation rate after sequestrectomy in our pooled data. On the other hand, patients with small annulus defect may be better candidates for sequestrectomy.

Symptoms improvement and patients’ satisfaction were also analyzed in our study. Patients who received sequestrectomy suffered significant less low back pain but equivalent sciatica. Further, the incidence of postoperative analgesic usage was significant lower in sequestrectomy group at both short-term (<1 year) and long-term (>1 year) follow up. Therefore, a better functional improvement and satisfaction occurred in sequestrectomy group. As a conventional procedure without curettage, the sequestrectomy gave rise to significantly less loss of disc height and endplates degeneration [37], which may reduce “failed back syndrome” as a result of better intervertebral stability and less spondylosis [8,9]. Disc degeneration accompanying with facet pathology gains the risk of recurrent low back pain after discectomy, in which aggressive disc resection and space curettage lead to an aberrant axial force distribution to the annulus fibrosis and facet joints [8,9,38,39].

There are several limitations of this meta-analysis. First of all, in lack of RCTs, prospective and retrospective comparative studies were also included in our research. Methodological defects have been found in some of these studies, including non-contemporary design, different follow-up time, unpaired baseline characteristics, high rate of loss to follow up, no prospective collection of data, non-blinding evaluation and patients’ selection. Except for the RCT conducted by Thome, C [13], sequestrectomy was applied to a restricted patients’ subgroup, whose annular defect was small and herniated fragment was extruded or sequestrated. Secondly, continuous outcomes were provided in form of only mean values without SD in some studies and couldn’t be involved in meta-analysis. This may produce bias. Thirdly, one suspected study was excluded since the full-text wasn’t available. Despite these weaknesses, our meta-analysis can still provide some value for clinical reference.

## Conclusion

According to our pooled data, sequestrectomy entails equivalent reherniation rate and complications compared with discectomy but maintains a lower incidence of recurrent low back pain and higher satisfactory rate. High-quality prospective randomized controlled trials are needed to firmly assess these two procedures.

## Supporting Information

S1 PRISMA Checklist PRISMA checklist. (DOC)
